# The Analysis of Stress Raisers Affecting the GFRP Strength at Quasi-Static and Cyclic Loads by the Theory of Critical Distances, Digital Image Correlation, and Acoustic Emission

**DOI:** 10.3390/polym15092087

**Published:** 2023-04-27

**Authors:** Dmitrii Lobanov, Andrey Yankin, Maksim Mullahmetov, Ekaterina Chebotareva, Valeriya Melnikova

**Affiliations:** 1 Center of Experimental Mechanics, Perm National Research Polytechnic University, 614990 Perm, Russia; m.mullahmetov59@gmail.com (M.M.); cem.chebotareva@mail.ru (E.C.); cem.melnikova@mail.ru (V.M.); 2 Department of Mechanical and Aerospace Engineering, School of Engineering and Digital Sciences, Nazarbayev University, 010000 Astana, Kazakhstan; andrei.iankin@nu.edu.kz

**Keywords:** composite materials, theory of critical distances, fatigue tests, acoustic emission

## Abstract

The purpose of this work is to analyze the stress-raisers that affect the tensile strength and fatigue resistance of GFRP parts using the point and line methods of the theory of critical distances (TCD) to obtain a quantitative measure of the defect size that can be tolerated by the composite before it fails. In the course of the work, a method combining TCD and the Weibull function was developed. In the course of the work, GFRP structural fiberglass for electrical purposes was tested under uniaxial quasi-static and cyclic loading with digital image correlation (DIC) and acoustic emission (AE), as well as a numerical simulation of deformation. The studied specimens were plain (without a stress-raiser) and notched (V-shaped) with different notch root radii and depths. The results were used to determine the material critical distances. In this case, two approaches to TCD were used: line (LM) and point (PM) methods. To analyze the experimental results, finite element modeling was applied using the ANSYS software package. As a result, the linearized maximum principal stresses were obtained on the central line passing through the top of the stress raiser. Thus, the values of the critical distances of the material were determined by PM and LM. Based on the data obtained, the sizes of permissible defects in the studied fiberglass were established that do not affect the tensile and fatigue strength of the material. The paper illustrates the cumulative energy, peak amplitudes, and distributions of the frequency of the spectral maximum of acoustic emission signals obtained after the destruction of specimens by fatigue test. Evolutions of deformation fields on the specimen surface were recorded using a Vic-3D contactless optical video system and the DIC.

## 1. Introduction

New technologies and materials are expected to both improve products’ performance and reduce material consumption, which entails increased reliability requirements under complex thermo-mechanical influences. When introducing composite materials to critical structures, it is important to analyze the conditions of the destruction and survivability of products. The issues in predicting the bearing capacity of structures and products made of composites remain unresolved. Therefore, it is necessary to understand how the locations and geometric parameters of defects influence the material and its mechanical properties [1,2,3,4,5,6]. Composite materials are widely used in various fields, which determines the importance of their study. At the same time, composites are used both separately and in combination with other materials: concrete, metals, plastics, etc. [7,8,9,10,11].

Many defects can arise throughout manufacturing processes: cracks, chips, scratches, dents, impact defects, air macro inclusions, warpage, non-gluing, and others. All these defects may significantly reduce the static and fatigue resistance of the structures. There are plenty of methods that affect the severity of the defects, for example, a proper control of the manufacturing process using artificial intelligence, varying the parameters of the manufacturing process, etc. Aside from process optimization, post-processing may also influence the defects described above, for instance, local repairs and machining. Thus, the defects can be minimized by process optimization and/or post-processing but not fully eliminated.

When predicting the effect of stress concentrators, various methods can be applied, for example, the “hot spot” method, based on the maximum peak stress at the base of the notch; however, as evidenced in practice, this is too conservative. To solve this problem and improve the analysis results, Neuber and Peterson [12,13] proposed the linear method (LM) and the point method (PM), respectively, with the idea that a critical volume of the must be subjected to a critical stress for fatigue failure to occur. Subsequently, Tanaka [14] and Lazzarin et al. [15] generalized all the proposed methods. Taylor also introduced a description of a family of related methods such as the critical distance theory (TCD) [16]. A common feature of this theory is the use of a parameter (critical distance), which depends on the material. TCD has been successfully applied to the strength/life prognosis of a wide range of different materials, such as metals, alloys, composites and concrete.

The classical TCD assumes that the strength of the notched components can be estimated by analyzing the linear-elastic stress field in the vicinity of the stress concentrator. Often, this approach is used for fatigue loading, but it is also applicable to quasi-static loading [17,18,19,20,21].

Several different approaches have been adopted using TCD to analyze experimental data and predict the safe parameters of internal defects. In principle, the required parameters (critical distance and stress) can be obtained from the results of experiments carried out for only two types of specimens; for example, a plain specimen (without a notch) and a specimen with a notch, or on two specimens with different notches.

In conventional TCD, the critical distance is a material parameter that depends only on the asymmetry factor and the number of cycles to failure [22]. It was shown in [23] that defects, the size of which are much smaller than the critical distance, can be considered harmless for the fracture mechanism under consideration; however, such results require verification. Thus, TCD can be used to predict not only the mechanical behavior of bodies with stress concentrations but also to determine the critical defect size under various loading conditions. The values of the critical distance correlate with the microstructure of the material, so this approach could be extended to composite materials with different periodicity cells. Some alternative methods should also be noted: “injected crack” and “imaginary crack” models; approaches focusing on zones in front of the crack, nominal stress methods, methods of local stress–strain states, approaches based on weighted parameters, and others. More detailed reviews are presented in [16,24].

In the literature, there are often various modifications of TCD, methods and approaches to its use, aiming to obtain more accurate forecasts. Some of the modifications are presented, for example, in papers [18,25,26,27] for the cases of the elastic-plastic behavior of the material, the combined effect of complex geometries and process-inherent defects, etc. The articles by Lanning et al. [28], Yamashita et al. [29], and Wang et al. [30] should also be noted. These works focus on studying the effect of the size of the critical distance. At the same time, some problems are revealed when applying the method to small notches and large values of the loading cycle asymmetry coefficient.

The specificity of TCD allows it to be combined with various approaches: for example, with the strain energy density criterion [31] and multiaxial fatigue models [32,33]. Moreover, the TCD methods presented here do not consider the rigidity of the loading system, i.e., they predict the same result regardless of the load rigidity. Therefore, some approaches that consider post-critical deformations [34,35] may be also combined with TCD to overcome such an issue and enhance the resulting prognosis of the predictive model going forward.

At present, to analyze the inhomogeneity of inelastic deformation in materials, optical methods of experimental mechanics are increasingly being used [36,37]. Of the optical methods, the digital image correlation method (DIC) is the most relevant [37,38,39]. This method makes it possible to study the deformation and destruction of materials (metals, alloys, etc.), as well as to describe the processes that develop on the surface of the loaded specimens in a non-contact manner. Several scientific papers note the application of the correlation method to studies of the mechanisms of destruction of composites. To identify the types of destruction of composite laminates, the authors of Lala Bahadur Andraju and Gangadharan Raju [40] used the CCI method. The results obtained with this method have provided an insight into the development of the damage models to study the failure of composite laminates. A description of the types of damage to these materials using the optical CCI method is found in several other foreign works [41,42]. The work of Clifton H. Bumgardner et al. [43] studies the failure mechanisms of composites with a ceramic matrix. The authors noted the influence of the correlation processing on the accuracy of the results obtained during the CCI tests. The choice of correlation processing parameters is described in the works of Tretyakova T.V. [44], Strungar E.M. [39,45], and Sutton M.A. [46].

When analyzing the size and position of defects and the development of cracks, nondestructive testing methods are often used, e.g., the acoustic emission (AE) method. AE is a passive method for monitoring the propagation of cracks in materials. The occurrence of microcracks due to the load generates a surge of stress waves, which can be detected by AE sensors. The registration of acoustic emission events during tension of fiber-reinforced composites makes it possible to determine and track damage, including the moments of occurrence and progression of damage [47,48,49,50,51]. Many laminate failure models are based on static tension or bending and can be used to identify dominant damage types and further classify them by AE parameters. However, when it is difficult to estimate the destruction processes in advance, as in the case of fatigue mechanical loading, algorithms for clustering AE parameters have been applied. In the research, the authors use such clustering methods, such as k-means, c-means, self-organization by the Kohonen map (SOM) by Fourier spectra, artificial neural networks, and others [52]. The division of the energy of acoustic emission phenomena into clusters under cyclic loading makes it possible to determine the staging of the transition from damage accumulation to destruction.

The purpose of this work is to study the effect of stress concentration on the strength and fatigue life of GFRP using non-destructive testing methods such as acoustic emission registration and digital image correlation, check the applicability of TCD approaches (PM and LM) to predict the failure of GFRP parts, determine the critical distance values of the composite for a quantitative measure of the maximum defect size that does not affect the material resistance, and explore the effect of fatigue life and geometry features on the critical distance values of GFRP.

## 2. Material and Methods

### 2.1. Material

**Figure 1:**
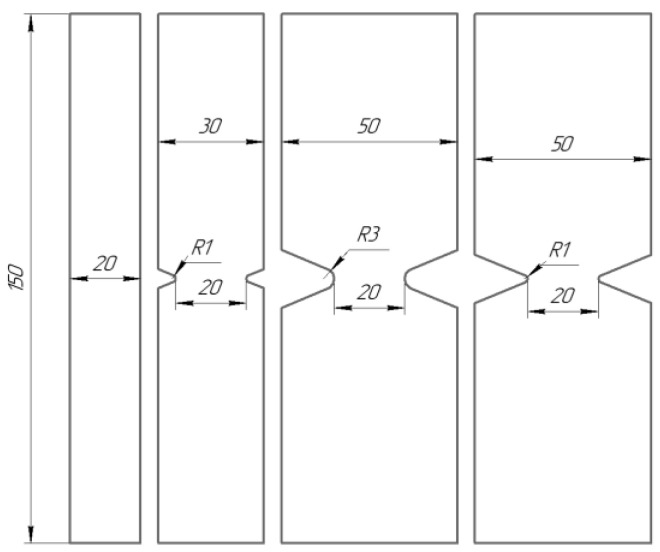
Specimens’ geometry.

The model layered polymer composite STEF (ST—fiberglass; EF—epoxy-phenol-formaldehyde or epoxy binder) was studied in this work. This is the laminated reinforced fiberglass obtained by hot-pressing of the fiberglass cloth impregnated with a thermoreactive compound based on combined epoxide and phenol-formaldehyde resins. Specimens without concentrators (in the form of strips) and specimens with a concentrator in the form of a V-shaped cut in the working part were made from a GFRP sheet 5 mm thick. Specimens of each geometry were cut in the warp and weft directions. Figure 1 shows the specimens. As specimens with stress concentrators, four varieties with different geometries were used: the depth of the notch and the radius of the apex of the notch was 15 mm and 3 mm (R3); 15 mm and 1 mm (R1), 5 mm and 1 mm (R1), respectively. In the article, the following naming system for specimens is adopted: Not. sp. 15 × 3, Not. sp. 15 × 1 Not. sp. 5 × 1.

Notch geometries were chosen to consider variations in the notch length and radius. This is necessary for the further checking of forecast methods to exclude the geometry influence of various stress-raisers on the result. Further in the article, the following naming system for specimens is adopted: Not. sp. 15 × 3, Not. sp. 15 × 1 Not. sp. 5 × 1.

Program and conditions of static and cyclic tests are presented of Table 1.

### 2.2. NDI Technique

To analyze the mechanisms of destruction during the test, the methods for recording the acoustic emission and digital image correlation were used.

The mathematical apparatus of the VIC-3D video system is based on the digital image correlation method (DIC). With correlation processing, it is possible to track the crack localization zone and its subsequent development on the specimen surface. The three-dimensional optical system Vic-3D (Correlated Solutions, Irmo, SC, USA) includes two digital cameras (sets with a resolution of up to 16.0 MP and a corresponding shooting speed of 3.3–4.8 Hz) [45].

**Figure 2:**
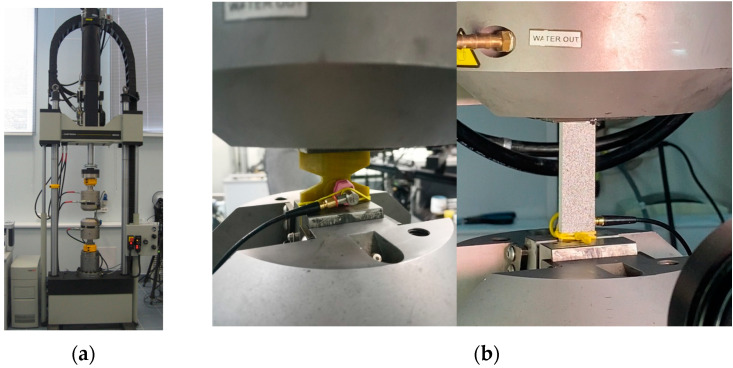
Instron 8802 test system (**a**) and a specimen with an acoustic emission sensor fixed in the grips of the test setup (**b**).

A continuous recording of the acoustic emission data was carried out using the multichannel AMSY-6 system from (Vallen System GmbH, The Acoustic Emission Company, Icking, Germany) from the beginning of the test to the destruction of the specimens. The AE144A piezoelectric transducer (Fujicera, Fujinomiya, Japan) (frequency range 100–500 kHz) (Figure 2b) and a 34 dB gain preamplifier (Vallen System GmbH, Icking, Germany) were used in the tests. The data sampling rate was 10 MHz. The sensors were attached to the specimens using Wacker Silicon vacuum silicone grease (DRAWIN Vertriebs-GmbH, Riemerling, Germany) and a mounting system.

Using the system for recording the acoustic emission signals, the dependences of the AE parameters on the number of cycles were obtained and visualized. The energy of AE signals, peak amplitudes, and peak frequencies were chosen as parameters. The energy parameter of the acoustic emission signals is presented as the cumulative energy obtained by summing the energy values of the signals for all previous intermediate time intervals; the cumulative energy shows the level of damage accumulation in the material.

### 2.3. Mechanical Tests

For testing static tension and tensile fatigue life, an Instron 8850 (Instron—division of ITW Limited, High Wycombe, UK) (100 kN) servo-hydraulic testing machine was used, as well as a set of measuring equipment for strain control (Vic-3D video system) and control of acoustic emission signals. Tensile tests were carried out at a moving grip speed of 2 mm/min.

To carry out fatigue tests, a program was developed based on the results of the static tensile testing of GFRP specimens of the corresponding geometries. For each type of specimen, the values of stresses in the working zone (in the region of the concentrator) were obtained.

For fatigue testing, a stress factor was introduced that reflects the ratio of the maximum stress realized per cycle to the tensile strength of the corresponding specimen (1):

k = σ/σ_b_ (1)

The tests were carried out at a cycle asymmetry coefficient R = 0.1, a frequency of 10 Hz with a sinusoidal cycle shape, at room temperature. The S-N curve functions are models, preferably on a ‘log–log’ or ‘lin–log’ scale, for a description of the phenomenon of fatigue life. Different S-N functions could be applied to various materials; for instance, the logistic function, Kohout–Vechet model, Basquin model, and Weibull model. Most of them are presented in other papers [53,54]. In this work, the Weibull model was utilized to describe the fatigue data as follows:

(2) $$\sigma_{\max}=\left(\sigma_{u}-\sigma_{\infty }\right)\exp[-\alpha \left(\log N\right){}^{\beta }]+\sigma_{\infty }$$

where *σ*_max_ is applied peak stress, *σ_u_* is ultimate tensile strength, $\sigma_{\infty }$ is fatigue limit, *N* is the number of cycles at failure, *α* and *β* are model fitting parameters.

### 2.4. TCD

**Figure 3:**
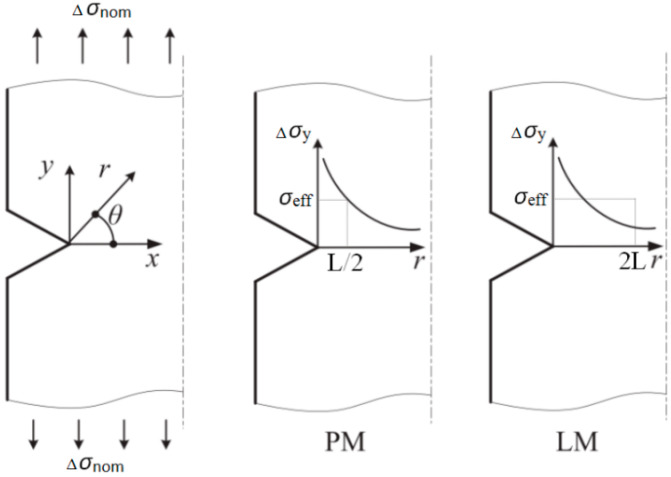
Line and point methods of the TCD.

The TCD method is essentially a linear elastic fracture mechanics (LEFM) approach, and, as such, is based on the elastic stress analysis of the notch or feature in question. The stress at a characteristic point or the average stress over a characteristic line in the vicinity of the notch is considered. Failure is predicted to occur when the stress range at the point (or averaged over the line) is equal to the stress needed to cause failure in a plain specimen at the same number of cycles. According to the topology type, TCD can be divided into the following categories, as shown in Figure 3.

According to the point method (PM):

(3) $$\sigma_{eff}=\Delta \sigma_{0}=\Delta \sigma_{y}\left(\theta =0,r=L/2\right)$$

According to the line method (LM):

(4) $$\sigma_{eff}=\Delta \sigma_{0}=\frac{1}{2L}\int_{0}^{2L}\Delta \sigma_{y}\left(\theta =0,r\right)dr$$

where Δ*σ_y_*(*r*) is the elastic stress range as a function of distance ‘*r*’ from the notch root for the notched specimen, Δ*σ*_0_ is the maximum stress values causing fatigue failure in the given number of cycles for the plain specimen, *L* is the critical distance. For the particular case of infinite life (i.e., the fatigue limit), the critical distance can be defined as

(5) $$L=\frac{1}{\pi }{\left(\frac{\Delta K_{th}^{}}{\Delta \sigma_{0}^{}}\right)}^{2}$$

where Δ*K_th_* is the threshold stress intensity range for fatigue crack growth in the material.

**Figure 4:**
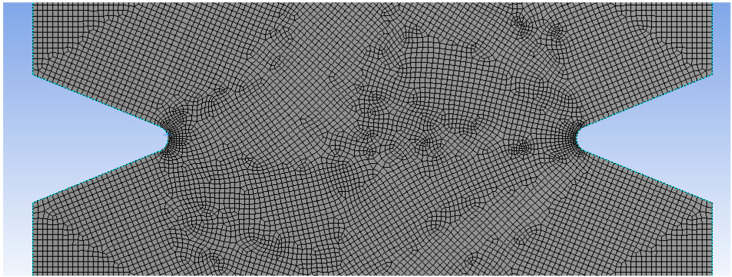
Example of FE mesh.

In order to determine the stress–distance curve, 2D finite element (FE) models were made using the commercial software ANSYS (Ansys workbench 2019 R3). The specimens were designed as a single solid structure. Mesh parameters included 119,196 elements and 515,994 nodes. Plain 145 finite elements with 8 nodes and quadratic approximation were used. Figure 4 shows an example of an FE mesh used to model the notched specimens. By doing so, a refined mesh of about 0.025 mm element size in the critical region of the notch tip was applied. The size of the finite elements was selected in such a way that at least 5 finite elements were located within the critical distance [55]. These were sufficient to give accurate stress–distance information in the critical regions.

Material properties (Young’s modulus and Poisson’s ratio) were selected from the tensile tests. One of the specimen edges was restrained and a given vertical load was applied to another edge. The applied vertical load was calculated as a product of the maximum stress and minimum cross-section area. The maximum stress corresponded to the notched specimens with a root radius of 1 mm and notch depth of 15 mm, whereas *σ*_0_ corresponded to the plain specimens.

For the implementation of the TCD analysis, the linearized maximum principal stress was recorded as a function of distance from the notch tip along a line given by the extension of the notch: the stress–distance curve, *σ_y_*(*r*). Given this stress–distance curve, and the appropriate plain-specimen fatigue strength, two values for *L* can be found using the PM and LM, respectively.

## 3. Results and Discussion

### 3.1. Mechanical Test

**Figure 5:**
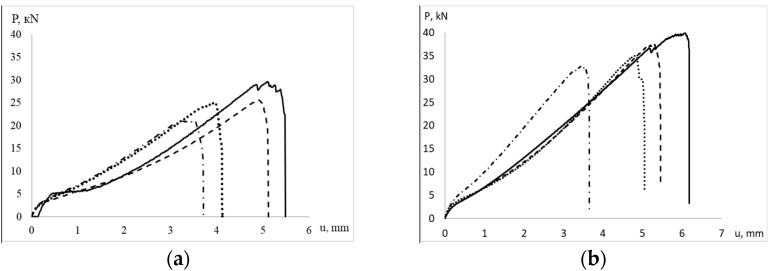
Loading diagrams of specimens. (**a**) weft direction, (**b**) warp direction: the dotted line is Not.sp. 15 × 1, the solid line is the plane specimen, the dot–dash line is Not.sp. 15 × 3, the dashed line is Not.sp.5 × 1.

For each type of specimen, quasi-static tensile tests were carried out to obtain the ultimate load values. Loading diagrams are shown in Figure 5. The data are shown in Table 2.

**Figure 6:**
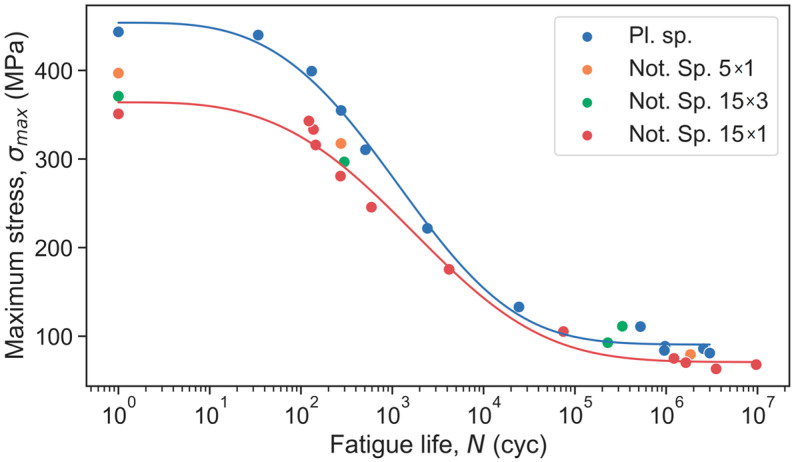
Fatigue results for plain and notched specimens.

The results of the fatigue test were expressed in terms of the maximum stress versus fatigue life diagram, as shown in Figure 6. Additionally, this diagram displays the results of the tensile tests as maximum stress at one load cycle that corresponds to the ultimate tensile strength. Overall, the experimental results show that the maximum stress values the notched specimens can withstand are less than those of the plain specimens. The Weibull fitting parameters were obtained only for two types of specimen: plain and 15 × 1 notched (Table 3). As one may see from Table 3, *α* and *β* values for plain and 15 × 1 notched specimens do not change significantly. This means that the only difference is related to *σ_u_* and *σ_∞_* values. In this case, the ultimate tensile strength values may be considered the fatigue strength at one cycle while the fatigue limit values are obtained at an infinite number of cycles.

### 3.2. TCR Application

**Figure 7:**
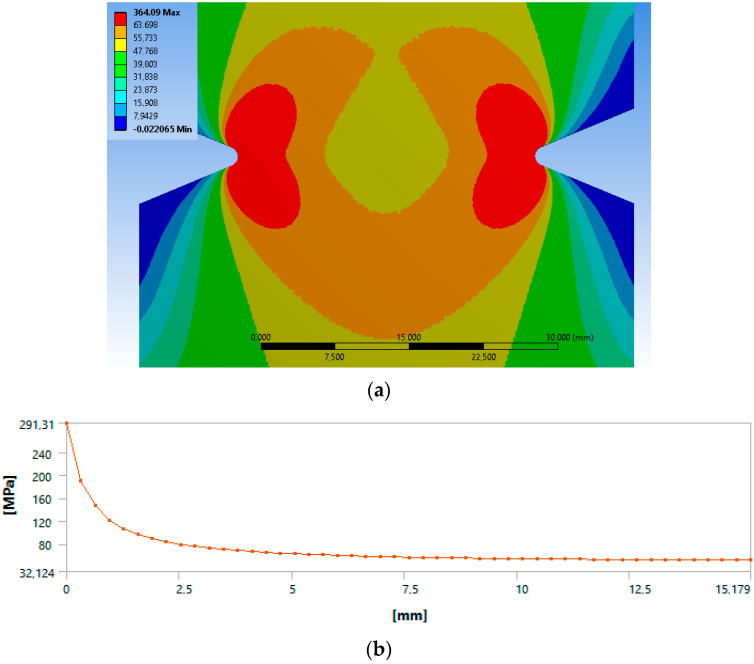
Results from the FEM for the notched specimen: (**a**) contours of maximum principal stress in MPa and (**b**) linearized maximum principal stress in MPa.

A numerical study of all the used specimen geometries was conducted. Figure 7 illustrates, as an example, the FEM output for the 15 × 1 notched specimen. Thus, utilizing the linearized maximum principal stress as a stress–distance curve, as well as *σ_u_* and *σ_∞_* values from Table 3, the critical distances were calculated at one and an infinite number of cycles:

- L_PMu_ = 2.60 mm (at *N* = 1 cycle);
- L_PM∞_ = 2.47 mm (at *N* = ∞ cycles);
- L_Lmu_ = 2.19 mm (at *N* = 1 cycle);
- L_LM∞_ = 2.05 mm (at *N* = ∞ cycles).

The fatigue life ‘*N*’ negligibly affects the critical distance values. In other words, a minimal change in L is shown as fatigue life increases. The difference is about 5% and 7% for PM and LM respectively. Therefore, the hypothesis was formed that the critical distance could be considered as a constant at the fatigue life and equal to its value calculated at one cycle, i.e., under the tensile test.

Therefore, critical distances of 2.60 mm and 2.19 mm were selected for PM and LM, respectively, to predict the stress values of specimens in both warp and weft directions. TCD predictions were then developed using both PM and LM methods, and the results are presented in Table 4. It should be noted that the most accurate values of ‘L’ may be calculated by averaging the values from all the used specimens or by calculating the Weibull function in case there is a high dependence of the critical distance on the fatigue life:

(6) $$L_{\mathrm{PM}}=\left(L_{\mathrm{PMu}}-L_{\mathrm{PM}\infty }\right)\exp[-\alpha \left(\log N\right){}^{\beta }]+L_{\mathrm{PM}\infty }$$

(7) $$L_{\mathrm{LM}}=\left(L_{\mathrm{LMu}}-L_{\mathrm{LM}\infty }\right)\exp[-\alpha \left(\log N\right){}^{\beta }]+L_{\mathrm{LM}\infty }$$

where *α* and *β* are model fitting parameters from Table 3. This is a different approach to that which was applied in this study, whereby average L values were taken from the most severely notched (15 × 1 mm) specimen under quasi-static tensile tests only. Nevertheless, it is worth noting that other options are also available. Going forward, further studies of various modifications could be worth exploring to compute the most accurate TCD forecasts. Error values were calculated as follows: Error = 100 (Pred.Stress − Exp.Stress)/Exp.Stress %.

**Figure 8:**
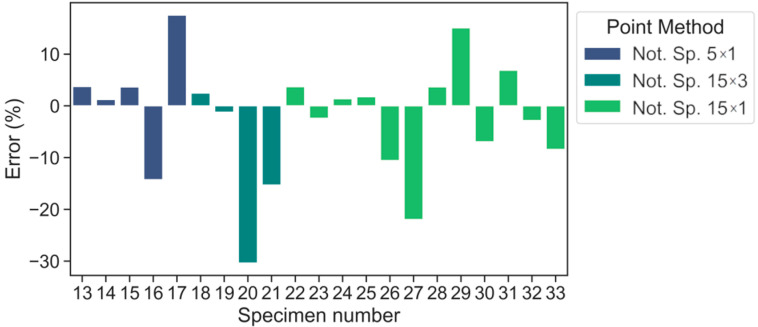
Error values according to PM predictions (warp direction).

**Figure 9:**
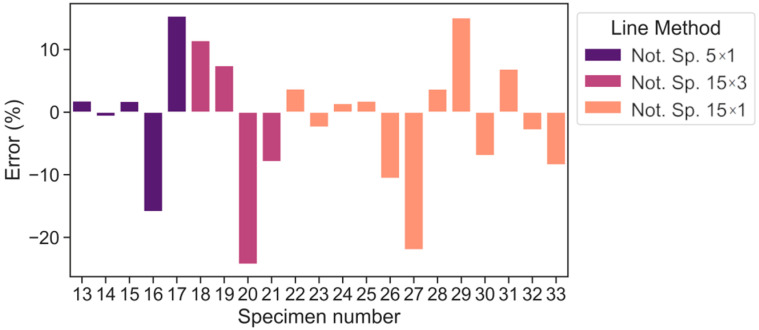
Error values according to LM predictions (warp direction).

**Figure 10:**
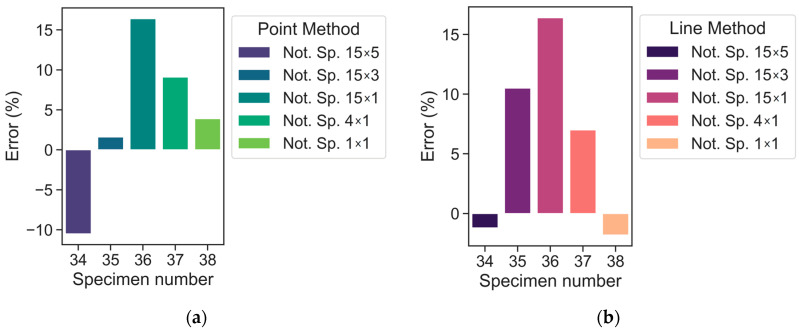
Error values according to PM (**a**) LM (**b**) predictions (weft direction).

On account of the error values (%), Figure 8 and Figure 9 illustrates the advantage of LM over PM in terms of maximum values. The maximum error is within ±30.4% for PM and ±24.3% for LM (warp direction). By doing so, the average absolute errors are equal to 8.4% and 8.2% for PM and LM, respectively. In the case of the weft direction (Figure 10), the maximum error values are within ±16.4% and the average absolute errors are equal to 8.3% and 7.4% for PM and LM, respectively. A maximum average absolute error of 12.8% was obtained using the LM method. Maximum errors of the selected data points exceeded 20% only for the 20th (15 × 3) and 27th (15 × 1) notched specimens. Overall, LM is preferable to PM in this scenario.

It should be noted that although PM and LM show acceptable predictive ability maximum errors for the 20th (15 × 3) and 27th (15 × 1) notched specimens exceed ±20%, if one looks at these test points in Figure 6, one may notice that they are slightly away from the main point group. Consequentially, they may be considered outliers. However, even for these tests, the fatigue stress can be predicted with errors of 24.3% according to LM.

To further assess the predictive accuracy of both TCD approaches, the error of the Weibull function can be calculated. By performing such an analysis, the error values of the Weibull curve may be regarded as experimental errors. Thus, discrepancies between the experimental data points for the plain specimen set and the subsequent Weibull curve that was formed are accounted for. Then, TCD errors for both PM and LM approaches may be compared to the experimental error.

The maximum experimental error is equal to −17.4%. Therefore, it is supposed that any TCD error that falls within this range (±17.4%) is identified as a satisfactory one. Hereby, the TCD errors of most tests are within ±17.4%, except for two experiments for LM (specimens with marks 20, 27 from Table 4) and three experiments for PM (specimens with marks 17, 20, 27 from Table 3). It can be assumed these experiment points are outliers and concluded that TCD mostly provides acceptable predictions. In addition, it should be noted that if the notch/defect sizes (depth/length) are significantly less than the material critical distance, one could neglect this notch/defect in accordance with the paper [18].

In summary, the tensile strength values ‘σu’ of plain and 15 × 1 notched specimens (Table 2) were chosen as a training set while other experiments for notched specimens of various stress concentrations were considered as a test set for model validation. The error values presented in this section were acceptable, testifying to the negligible effect of notch geometry and fatigue life on the critical distance values.

The TCD is commonly utilized to predict the failure of a part due to the presence of stress-raisers caused by cracks, defects, or geometry features. The TCD provides a quantitative measure of the size of a defect that can be tolerated by a material before it fails [23]. If the notch/defect sizes (depth/length) are significantly less than the material critical distance, one could neglect this notch/defect.

### 3.3. Acoustic Emission

We obtained and visualized the dependencies of the AE parameters on the number of cycles before the destruction of the specimens using the system for recording acoustic evaluation signals. The energy of AE signals, the number of signals, peak amplitudes, and peak frequencies were chosen as parameters.

**Figure 11:**
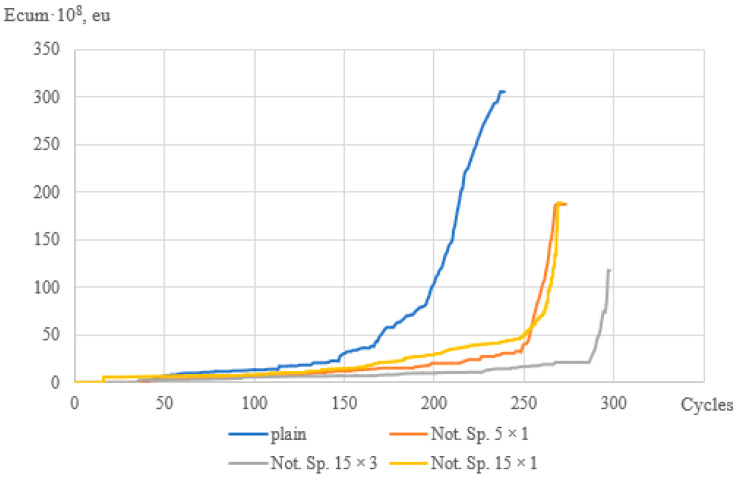
Diagrams of the cumulative energy of signals vs. the number of cycles (k = 0.8).

Diagrams of the dependence of the cumulative energy of AE signals on the number of cycles to failure of fiberglass specimens of various geometries are shown in Figure 11. For specimens at a load level of k = 0.8, different values of the cumulative energy could be noted depending on the geometry of the specimens. For the specimens without the stress concentration, the level of the cumulative energy is 1.5–2 times higher than for the specimens with the stress concentration. For the specimens with the stress concentration, a sharp increase is observed at 92–96% of the completed cycle, while for the specimens without the stress concentration, this value is lower by 10–15%, and the graph looks smoother. This may indicate that the accumulation of damage in the specimens with different geometries occurs differently with an approximately equal number of cycles and equal load.

**Figure 12:**
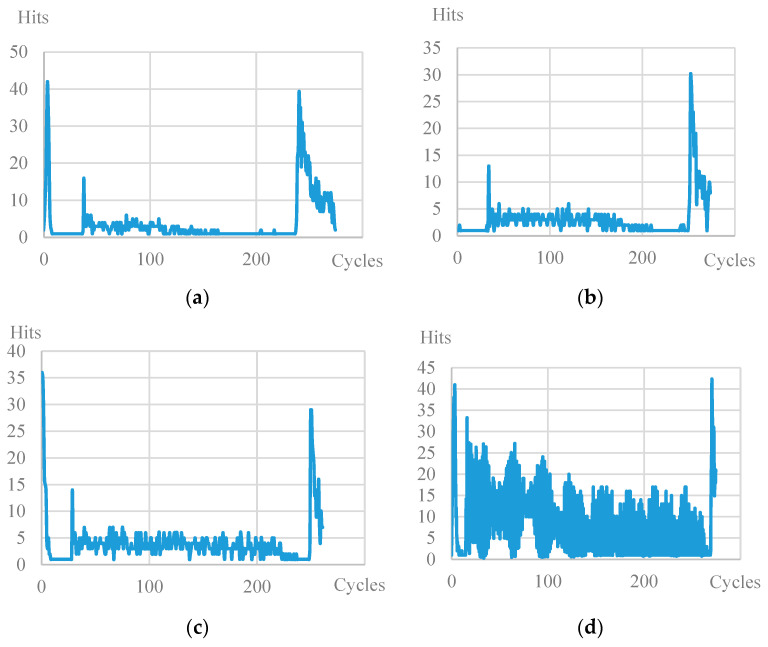
Diagram of the number of signals vs. the number of cycles (k = 0.8): (**a**) plain, (**b**) not. Sp. 5 × 1, (**c**) not. Sp. 15 × 3, (**d**) not. Sp. 15 × 1.

Figure 12 shows the dependencies of the number of signals on the number of cycles of the specimens with the same ratio of maximum load to the ultimate strength of the material k = 0.8. A characteristic surge at the beginning of the test and the end upon failure is observed for all types of specimen. However, for specimen 15 × 1, throughout the entire test, a high level of signal values can be seen compared to the specimens of other geometries. From the values of the energy parameter described above, one concludes that the signals emitted by the material had low energy values, although their number is higher than other types of specimen.

**Figure 13:**
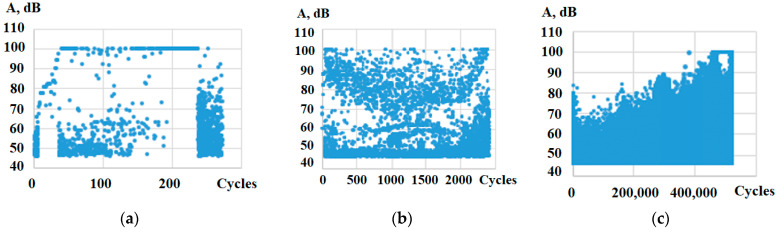
The diagram of peak amplitudes vs. the number of cycles for the plain specimens: (**a**) k = 0.8, (**b**) k = 0.7, (**c**) k = 0.25.

The amplitude-to-cycle ratio diagrams (Figure 13) show that, with a small number of cycles, the amplitude of the AE signals reached a maximum as early as the start of the test (Figure 13a,b), and high values of the amplitude were retained until specimen failure. The process of damage accumulation has a more staged nature, with an increase in the number of cycles; the amplitude reaches peak values before destruction. The material in this case “adjusts” to small loads, k = 0.25 (Figure 13c). However, the value of the peak amplitudes was recorded above 75 dB in all the tests, which indicates similar failure processes in the material [56].

**Figure 14:**
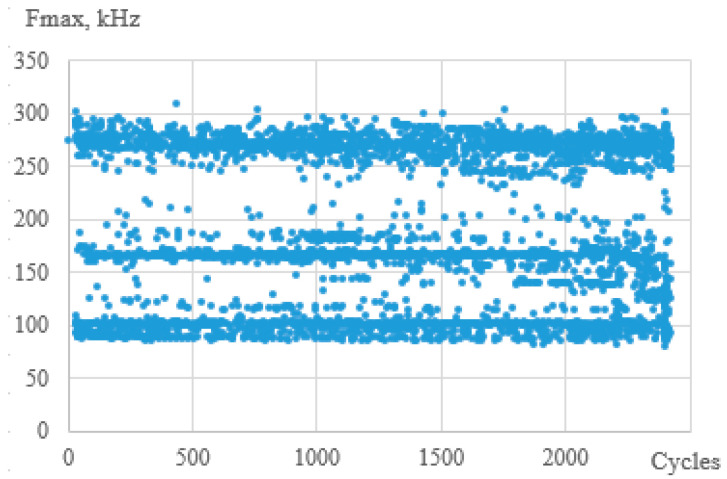
Typical diagram of peak frequencies vs. number of cycles.

A typical plot of the dependence of the peak frequencies on the number of signals is shown in Figure 14. Three frequency ranges are shown: from 100 to 140 kHz, from 160 to 200 kHz, and from 220 to 320 kHz. The authors believe that these ranges characterize failure mechanisms such as matrix cracking, delamination, and fiber rupture, respectively [57]. In this representation, peak frequencies are present for specimens with different geometries in all three ranges. This emphasizes the need to analyze the histograms of the distribution of the peak frequencies from the number of signals.

**Figure 15:**
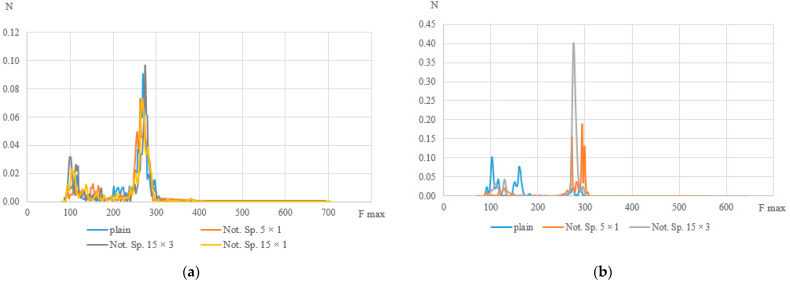
Histograms of frequency distribution of the spectral maximum (**a**) k = 0.8, (**b**) k = 0.25.

**Figure 16:**
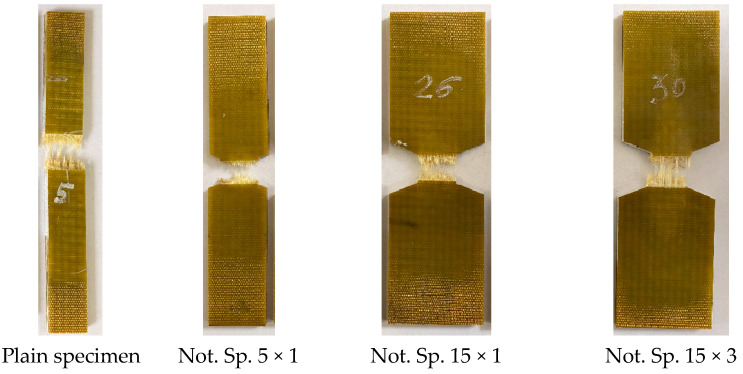
Fracture of specimens with various notch geometries.

The peak frequency distribution histograms (see Figure 15) show that, at k = 0.8, specimens with various geometries have frequencies distributed in the same ranges (as mentioned above, for low-frequency and high-frequency ranges, there are also signals in the middle-frequency range). However, the geometry of the specimens affects the mechanisms of failure in the material with an increase in the number of cycles. With a decrease in the relative level of stress and, accordingly, an increase in the number of cycles, solid specimens recorded signals in all frequency ranges without a clear predominance. At the same time, for specimens with stress concentrators, the number of high-amplitude signals ranged from 20 to 40% of the total at k = 0.25, when, at k = 0.8, this value does not exceed 10%. It is worth noting that the high-frequency range stands out on most specimens. Frequencies with these values have the maximum number of signals in relative units, from which we can conclude that the rupture of fibers in the material is the main mechanism of failure during cyclic tests, which can be seen in the photographs of the specimens after their failure (see Figure 16).

### 3.4. Digital Image Correlation

**Figure 17:**
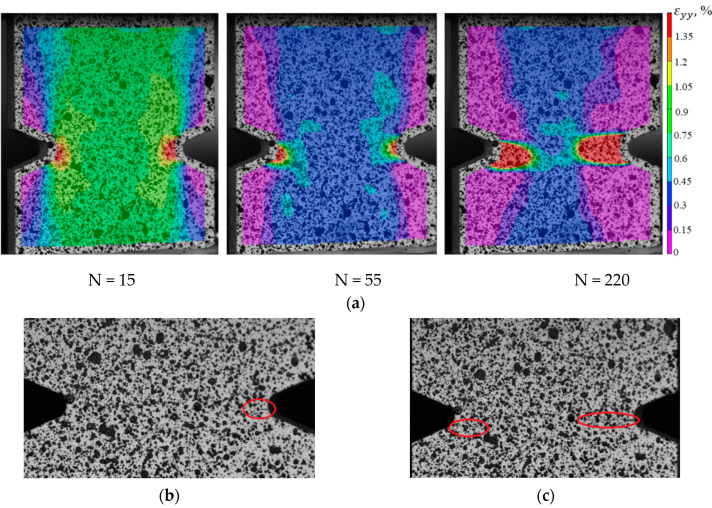
(**a**) Evolution of inhomogeneous fields of longitudinal deformations on the sample surface Not. sp. 5 × 1; (**b**) the photograph of the sample at N = 15; (**c**) the photograph of the sample at N = 220.

**Figure 18:**
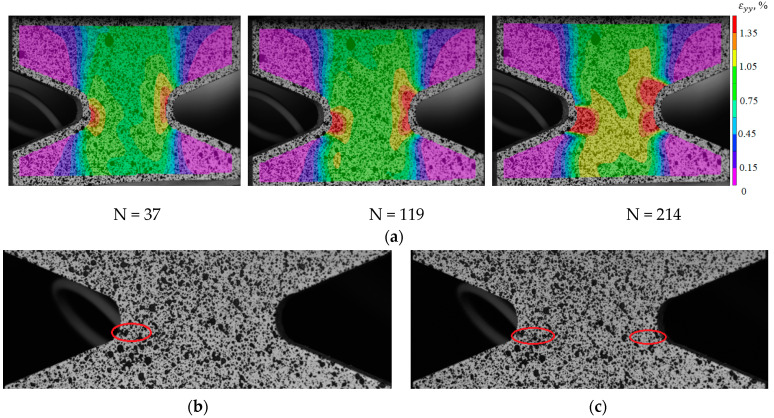
(**a**) Evolution of inhomogeneous fields of longitudinal deformations on the sample surface Not. sp. 15 × 3; (**b**) the photograph of the sample at N = 37; (**c**) the photograph of the sample at N = 214.

**Figure 19:**
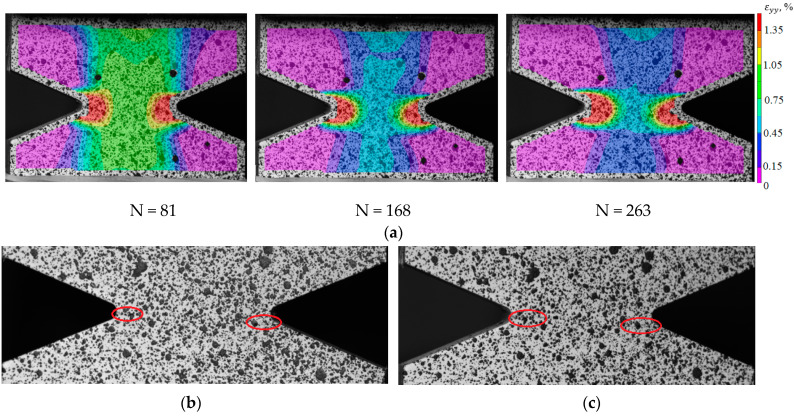
(**a**) Evolution of inhomogeneous fields of longitudinal deformations on the sample surface Not. sp. 15 × 1; (**b**) the photograph of the sample at N = 81; (**c**) the photograph of the sample at N = 263.

**Figure 20:**
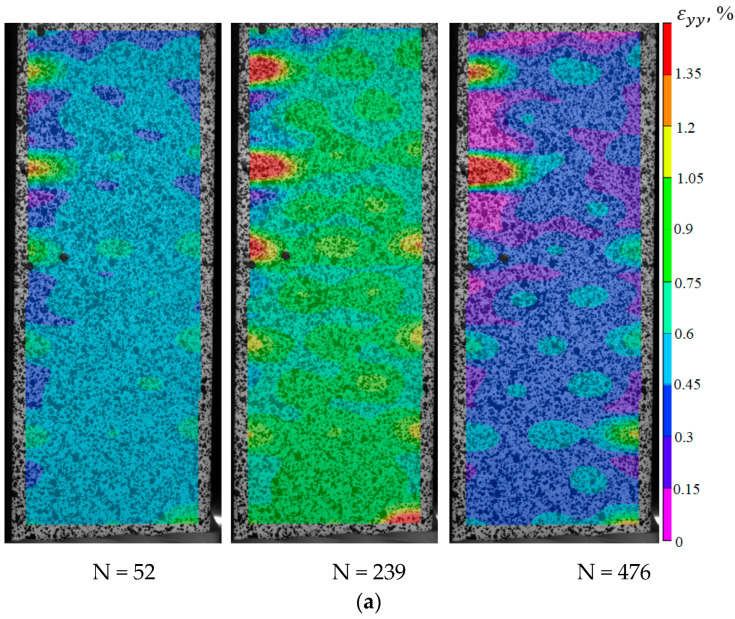

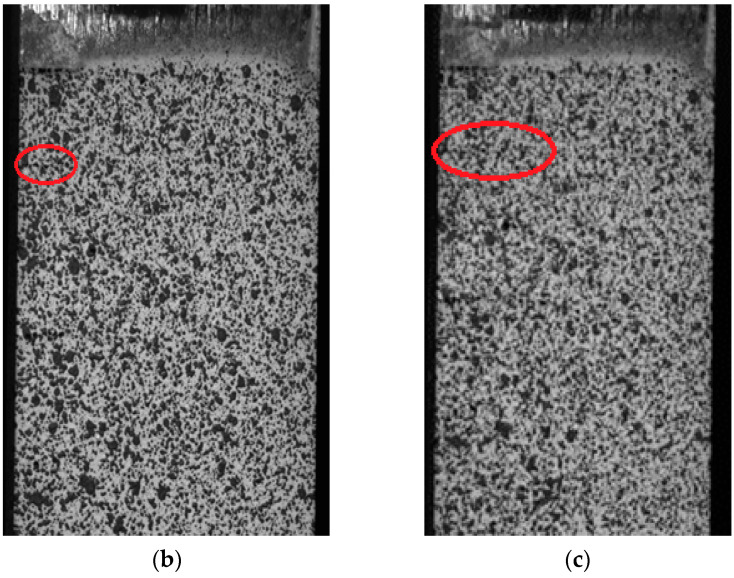
Evolution of inhomogeneous fields of longitudinal deformations on the specimen surface without stress concentrators (**a**); the photograph of the specimen at N = 239 (**b**); the photograph of the specimen at N = 476 (**c**).

To identify the fracture mechanism of specimens using a three-dimensional optical system Vic-3D, the evolution of longitudinal strain fields is constructed. With the help of the correlation analysis, it was possible to fix the beginning of the localization corresponding to a certain point in time and a certain cycle. For the specimens with V-shaped notches in the central part, the moment of crack-growing is recorded at the notch tip. For deformation patterns obtained using the Vic-3D software, images of the working area at the moment of loading the specimens are illustrated as an example. These pictures correspond to the crack growth start (the red circle marks the cracks) and the last photograph in the evolution of inhomogeneous longitudinal deformation fields, the time point, and the cycle (see Figure 17b,c, Figure 18b,c, Figure 19b,c and Figure 20b,c).

Thus, it was possible to fix the primary occurrence of fatigue cracks in the specimens without a cutout and with a cutout of a different geometry under cyclic loading. For specimen Not. sp. 5 × 1 (see Figure 17), the initial occurrence of the fatigue crack was recorded at the 15th loading cycle, which is approximately 6% of the total number of cycles before failure. For Specimens Not. sp. 15 × 3 and Not. sp. 15 × 1 (see Figure 18 and Figure 19), the initial appearance of the fatigue crack was recorded at 37 and 81 cycles, respectively, which was 30% and 17% of the total number of cycles to failure. For the plain specimen (see Figure 20), the occurrence of the fatigue crack on the surface was recorded at cycle 239, which was 50% of the total number of cycles before failure.

## 4. Conclusions

An analysis of the acoustic emission data shows that the geometry of the specimens affects the mechanisms of the material failure with an increase in the number of cycles. However, this effect is observed only at low load levels. For the specimens with stress concentration, failure occurs mainly due to the destruction of fibers. All types of failures are observed for the specimens without a stress concentration: matrix failure, fiber failure, and delamination. The results suggested that, at high load levels, the geometry of the specimens and the presence of the stress concentration do not significantly affect the nature of the failure mechanism.

An analysis of the strain fields obtained using the Vic-3D system showed the dependence of the moment of crack initiation on the type of the stress concentrator. These data are consistent with the cumulative energy calculated when processing the acoustic emission signals. The higher the stress concentration factor, the earlier the crack growth starts. At the same time, with an increase in the stress concentration factor, the total cumulative energy of AE signals decreases. This suggests that, in case of a later onset of crack growth, many internal defects are formed in the specimen, which do not affect the occurrence of the main crack or the strength of the specimen.

Additionally, a method was developed and applied that combines TCD (PM and LM) and the Weibull function to predict the fatigue resistance of the STEF composite part with a stress-raiser. The TCD is a science concept that is commonly used in industry to predict the failure of a part due to the presence of a stress concentration caused by a crack, flaw, or geometry feature. Analysis of the critical distance parameter ‘L’ showed no significant relationship between it and fatigue life. The critical distance values varied by less than 7% for both methods. Therefore, the ‘L’ parameter was considered as a constant at the fatigue life and equal to its value calculated under the tensile test: 2.60 mm and 2.19 mm for PM and LM respectively. While both PM and LM techniques have a mostly satisfactory accuracy, LM demonstrates enhanced accuracy. The analysis confirms that the majority of model errors fall within experimental error margins. Overall, utilizing LM and Weibull models, one can reach ±24.3% strength prediction accuracy. The TCD provides a quantitative measure: any defect, crack, or notch is considered harmless with a length that is significantly less than the critical distance.

The results that were obtained can be utilized in several ways within the industry. One way is to implement quality control measures to establish the minimum acceptable defect size for materials, which guarantees that all parts meet the required level of quality. Another application is in component design, where the information can be utilized to create components that are resilient under stress, ensuring their longevity. Lastly, the results can be utilized for inspection and maintenance purposes to determine the time at which a particular component should be inspected or replaced by monitoring the size of defects over time.

Additionally, TCD has a wide scope of application in research studies because it can be combined with different approaches and develop more accurate and complex models. It provides a framework for a better understanding of the mechanical behavior of materials and structures under loading conditions.

## Figures and Tables

**Table 1:** Program of static and cyclic tests.

No	Type Loading	Direction of Specimens Cutting	Specimens Type	Number of Tests	Test Conditions
1	Static tensile	Weft	Plain specimen	5	
			Not. Sp. 15 × 3	3	
			Not. Sp. 15 × 1		
			Not. Sp. 5 × 1		
2	Static tensile	Warp	Plain specimen	5	
			Not. Sp. 15 × 3	3	
			Not. Sp. 15 × 1		
			Not. Sp. 5 × 1		
3	Cycling tensile	Warp	Plain specimen	12	k = 0.99; 0.9; 0.8; 0.7; 0.6; 0.25; 0.2; 0.19; 0.18
			Not. Sp. 15 × 3	4	k = 0.8; 0.2; 0.25
			Not. Sp. 15 × 1	12	k = 0.98; 0.95; 0.9; 0.8; 0.7; 0.5; 0.3; 0.2; 0.18; 0.19; 0.21
			Not. Sp. 5 × 1	5	k = 0.8; 0.25; 0.2

**Table 2:** Ultimate load values for tested specimens.

Specimens Type	Ultimate Load, kN	
	Weft Direction	Warp Direction
Plain specimen	30.5	39.9
Not. Sp. 15 × 3	25.1	35.0
Not. Sp. 15 × 1	21.2	32.8
Not. Sp. 5 × 1	25.8	37.4

**Table 3:** Weibull fitting parameters.

Specimen Type	Model Parameters			
	*σ_u_*	*σ_∞_*	*α*	*β*
Specimen without notch	453.9	90.6	0.0152	3.42
Notched specimen 15 × 1	364.1	70.8	0.0146	3.29

**Table 4:** Predicted fatigue stress values according to PM and LM for each specimen data set.

No.	Fatigue Life (cyc)	Exp. Stress (MPa)	Pred. PM Stress (MPa)	Pred. LM Stress (MPa)	PM Error (%)	LM Error (%)	Specimen	Fiber Orientation
13	1 *	397.0	411.8	404.1	3.7	1.8	Not.Sp. 5 × 1	warp direction
14	274	317.6	321.5	315.5	1.2	−0.7	Not.Sp. 5 × 1	
15	1,844,698	79.4	82.3	80.8	3.7	1.7	Not.Sp. 5 × 1	
16	229,315	99.2	85.1	83.5	−14.3	−15.9	Not.Sp. 5 × 1	
17	10,000	119.1	140.0	137.4	17.5	15.3	Not.Sp. 5 × 1	
18	1 *	371.0	380.2	413.4	2.5	11.4	Not.Sp.15 × 3	
19	298	296.8	293.2	318.8	−1.2	7.4	Not.Sp.15 × 3	
20	331,000	111.3	77.5	84.3	−30.4	−24.3	Not.Sp.15 × 3	
21	229,315	92.8	78.6	85.4	−15.3	−7.9	Not.Sp.15 × 3	
22	1 *	351.0	364.1	364.1	3.7	3.7	Not.Sp.15 × 1	
23	145	315.9	308.4	308.4	−2.4	−2.4	Not.Sp.15 × 1	
24	271	280.8	284.7	284.7	1.4	1.4	Not.Sp.15 × 1	
25	591	245.7	250.0	250.0	1.8	1.8	Not.Sp.15 × 1	
26	4193	175.5	157.0	157.0	−10.6	−10.6	Not.Sp.15 × 1	
27	74,873	105.3	82.2	82.2	−22.0	−22.0	Not.Sp.15 × 1	
28	1,639,798	70.2	72.8	72.8	3.7	3.7	Not.Sp.15 × 1	
29	3,522,421	63.2	72.7	72.7	15.1	15.1	Not.Sp.15 × 1	
30	137	333.4	310.3	310.3	−6.9	−6.9	Not.Sp.15 × 1	
31	9,678,920	68.0	72.7	72.7	6.9	6.9	Not.Sp.15 × 1	
32	1,216,833	75.0	72.9	72.9	−2.8	−2.8	Not.Sp.15 × 1	
33	122	343.0	314.1	314.1	−8.4	−8.4	Not.Sp.15 × 1	
34	1 *	273.7	245.0	270.3	−10.5	−1.2	Not.Sp.15 × 5	weft direction
35	1 *	270.0	274.4	298.3	1.6	10.5	Not.Sp.15 × 3	
36	1 *	225.7	262.7	262.7	16.4	16.4	Not.Sp.15 × 1	
37	1 *	272.4	297.2	291.6	9.1	7	Not.Sp. 4 × 1	
38	1 *	310.7	322.7	305.1	3.9	−1.8	Not.Sp. 1 × 1	

* One cycle means that these experiments were conducted under quasi-static tensile loads.

## Data Availability

The data presented in this study are available on request from the corresponding author.
